# Examining Whether Risk Factors Influence Student Perceptions of Campus Sexual Violence

**DOI:** 10.5964/sotrap.14513

**Published:** 2024-11-27

**Authors:** Madison Wesenberg, Dakota D. Dickinson, Dana R. Haugen, Katerina Rubachuk, Sandy Jung

**Affiliations:** 1 Department of Psychology, MacEwan University, Edmonton, Canada; University of Lincoln, Lincoln, United Kingdom

**Keywords:** sexual violence, campus, risk factors, perpetration, student, perceptions

## Abstract

The present study examined university students’ perceptions of a campus sexual violence scenario, and specifically determined whether the presence of known risk factors for sexual perpetration would influence their views of an individual who caused harm. Two hundred and seventy-five student participants read a vignette that either included the present or absence of the following three risk factors: Frequent alcohol use, attitudes supportive of rape, and negative peer influence. The findings indicated that the risk factors did not have a significant relationship with students' risk perceptions, indicating that students’ perceptions of risk may not be influenced by the presence of known risk factors. The results suggest that students may use intuition or other non-scientific approaches when assessing instances of interpersonal violence, at least in campus settings. Implications for students, post-secondary institutional safety, and prevention of campus sexual violence are discussed.

## Highlights

- The study examines whether risk factors influence university students' perceptions about a campus sexual violence incident.
- University students who experience sexual violence are more likely to disclose to their peers and family and less likely to report such incidents to staff and faculty at their institution.
- Risk factors for sexual violence include rape myth acceptance, frequent alcohol use, and negative peer influence.
- Students' perceptions of a perpetrator's risk to commit further offences were not affected by the presence of known sexual violence risk factors.

Increasing attention to the prevalence of sexual violence at universities and colleges is evident in the media, through published literature, and by governmental agencies (Burczycka, 2020; Friedman, 2020; O’Boyle & Li, 2019). Much of the earlier attention focused on establishing victim support services and trauma informed approaches (Eisenberg et al., 2016; Khan et al., 2019), but greater resources have been directed at developing policies and ethical procedures to investigate these incidents (Henkle et al., 2020). What is well-known in the criminal justice field is that sexual assault reporting is only a fraction of what actually occurs (Cotter, 2021), and this unfortunate statistic is mirrored by what is seen on campuses, where sexual violence incidents are rarely reported (Burczycka, 2020). When they are reported, victims often disclose to informal supports, such as friends, peers, and family, over formal institutional supports, such as a sexual violence prevention office or other university staff (Mennicke et al., 2022). Victims have cited several barriers to formal reporting, such as fear of personal repercussions or that universities can’t or won’t respond (Burczycka, 2020). This latter point suggests that, regardless of the severity of the incident or the level of risk that a perpetrator poses to the post-secondary community, disciplinary decisions about the perpetrator may reflect the political climate and the institutional culture over the known risk factors that should be considered about campus safety.

Although all sexual assaults against post-secondary students, whether they occur on or off campus, should be investigated, a larger concern is about those incidents where the perpetrator may be at a higher risk to offend again, especially in the university context. This growing concern leads to the current study that examines whether students perceive an individual who has perpetrated sexual violence in a campus setting differently based on the presence of risk.

## Reporting of Campus Sexual Violence

In 2019, 71% of Canada’s post-secondary students experienced or witnessed unwanted sexual behavior in post-secondary settings (Burczycka, 2020). Less than 1 out of 10 women spoke about their experience to someone associated with the school, with most citing that they did not think the event was serious enough. Others stated they did not know what to do or indicated a mistrust of how the school would handle their complaint. Further evidence suggests that those who have experienced sexual violence face various barriers when it comes to non-reporting to formal sources.

One of the more common barriers is fear that no formal action will be taken following their disclosure (Lathan et al., 2023). Other identified barriers include the fact that survivors do not want family or friends to find out about the assault or they would rather avoid thinking or talking about the incident (Lathan et al., 2023). When victims do report their sexual assault, research has shown that victims are more likely to disclose their experiences to informal sources after unwanted sexual contact, unwanted sexual intercourse, and stalking, with rates ranging from 69% to 80%. When compared to the likelihood of disclosing these same behaviors to formal sources, rates of reporting drop and only 7% to 12% report to a post-secondary institution’s sexual violence office or other related services and supports (Mennicke et al., 2022).

Since most victims disclose to informal supports, which include student peers, it is important to examine how these informal sources of support perceive these disclosures. Of particular importance is how these student peers perceive the level of risk that the perpetrator poses to other students, as this information may propel them to either encourage the disclosing victim to formally report the assault or lead them to act in order to protect themselves and the victim. In the general literature, public perceptions of the risk that individuals who sexually offend pose have been examined (e.g., Harris & Socia, 2016; Levenson et al., 2007), and there seems to be a consistent finding that the public often overestimates the risk of these individuals and this estimation is often unrelated to empirical evidence (e.g., Lam et al., 2010; Levenson et al., 2007). However, less is known about how individuals who perpetrate sexual offences in a campus community are perceived by their peers. Before delving into these perceptions, it is important to explore what is known about campus sexual violence risk and what are risk factors that should be considered.

## Campus Sexual Violence Risk Factors

What is currently known about campus sexual violence risk is mostly based on two broad methodologies. Cross-sectional designed studies survey college and high school students, followed by correlational analyses between a variety of variables assessed and self-reported sexual aggression. There have also been longitudinal studies that have followed up with students over several years. Although there are many factors that have been empirically examined, it is important to recognize these empirical examinations are not likely exhaustive of all potential relevant factors.

### Cross-Sectional Studies

Most cross-sectional studies have focused on the relationship between various potential risk factors and self-reported sexual aggressive behaviors. For example, through an anonymous self-report survey of men from a large university, Abbey et al. (1998) found rape-supportive beliefs were positively correlated with the number of misperceptions, the likelihood of committing sexual assault, and the number of sexual assaults perpetrated. Higher than average alcohol consumption was also related to sexual misperceptions, which was correlated with sexual assault, but Abbey et al. also found that alcohol consumption was a mediating factor for this relationship. Carr and VanDeusen (2004) found that participants engaging in more sexually coercive behaviors also reported drinking more alcohol, and that sexual violence was related to alcohol use patterns. A study by Cleveland et al. (2019) examined the relationship between sociosexuality, heavy episodic drinking, and attendance at drinking venues, and sexual aggression. They found that alcohol venue attendance, rather than heavy episodic drinking, was found to be predictive of sexual violence perpetration across a five-semester time frame. A correlational study that was conducted by Forbes et al. (2006) found that college males who participated in aggressive high school sports (football, basketball, wrestling, and soccer) were more likely to engage in sexually coercive behaviors, as well as psychological and physical aggression with their partners. They also found that these individuals were more likely to endorse antisocial attitudes, including greater rape myth acceptance, sexism, hostility towards women, acceptance of violence, and homonegativity. Malamuth et al. (2021) found in their study that peer influence, extreme porn use, and adolescent delinquency were associated with contact sexual coercion and aggression.

### Longitudinal Studies

In addition to these cross-sectional approaches where data was collected at one point in time, longitudinal approaches to examine risk factors for sexual violence by university students have been employed. Thompson and her colleagues have conducted a series of studies over the years. Thompson et al. (2013) followed a sample of first-year university men with self-report surveys conducted at the end of every semester until graduation to predict sexual assault trajectories. Hostile masculinity, the number of sexual partners, alcohol misuse, and peer approval/pressure for persuading women into sex were identified as possible risk factors. The results found that believing in rape myths and having peers that condoned coercive techniques to engage in sex were consistent predictors of sexual assault, whereas alcohol misuse was not a significant predictor. Zinzow and Thompson (2015) followed up annually with male college students recruited in their first year and found that compared to non-perpetrators, single and repeat perpetrators engaged in significantly more risky behaviors, had rape-supportive norms, and scored high on rape myth acceptance. Comparing single and repeat perpetrators, repeat perpetrators scored higher on rape-supportive beliefs, peer attitudes toward coercive sex, and risky behaviors. In the same year, Thompson et al. (2015) published another longitudinal study and reported that those whose perpetration likelihood decreased, also showed decreases in sexual compulsivity, impulsivity, hostility towards women, rape supportive beliefs, peer approval of forced sex, peer pressure to have sex, and pornography use. Conversely, those in the group who had a high trajectory to perpetrate showed a larger increase in risk factors compared to those in the other groups.

A longitudinal study conducted by Abbey et al. (2012) examined the association between a variety of factors and patterns of sexual aggression over a 1-year period. They found that those who persisted (i.e., they committed sexual violence prior to and during the 1-year period) showed the most extreme scores on how they misperceived a woman’s sexual intent, while those who initiated sexual aggression during the period of study misperceived sexual intent more than non-perpetrators. A meta-analytic review by Steele et al. (2022) included longitudinal studies that gathered data from various higher education institutions. The purpose was to compare risk and protective factors for women at these institutions to prevent sexual violence. Some risk factors examined were alcohol consumption, rape myth acceptance, and peer approval of sexual violence. Peer approval was among the most significant risk factors associated with the perpetration of sexual violence. Alcohol consumption was also associated but not as strongly. Rape myth acceptance was more variable in its relationship with sexual violence perpetration.

### Review Studies

Several review studies have collated these findings either using systematic or meta-analytic approaches. Tharp et al. (2013) conducted a qualitative review of studies that included risk and protective factors for sexual violence, with the majority of samples coming from middle school, high school, and collegiate men and women. Among the risk factors for sexual violence, rape myth acceptance, peer influence, and alcohol consumption were identified as possible predictors, with rape myth acceptance seemingly predicting male-to-female sexual violence and alcohol consumption showing a linkage to increased perpetration of sexual aggression. An earlier meta-analytic study examined the effect sizes from 29 studies and the results showed that both fraternity membership and athletic participation were associated with increased rape myth acceptance and hypermasculinity, as well as sexually aggressive behaviors (Murnen & Kohlman, 2007). A more recent meta-analysis of 28 cross-sectional studies by Trottier et al. (2021) included both post-secondary and community samples and found that rape myth acceptance was positively associated with sexual coercion perpetration. In the same year, a systematic review of 28 studies was published by O’Connor et al. (2021) and reported that problematic attitudes (e.g., rape myth acceptance), peer influences, and alcohol and drug use were some of the most commonly researched risk factors for campus sexual violence perpetration.

### Summary

Seemingly consistent across these studies were variables that examined rape myth acceptance, substance usage, and peer approval or influence. Notably, these factors are seen in the criminal justice literature as risk factors (see Mann et al., 2010, for discussion of psychologically meaningful factors), and part of the central eight risk factors (Bonta & Andrews, 2017) as antisocial cognitions, problems with substance use, and antisocial associates.

## Rationale for the Present Study

For the present study, rape myth acceptance, alcohol venue attendance, and peer support were chosen as risk factors due to their strong empirical support. Past research has shown that these factors are predictive of sexual violence perpetration in campus, community, and offender samples (Mann et al., 2010; Tharp et al., 2013; Trottier et al., 2021; Zinzow & Thompson, 2015). They were also chosen as they are factors that could be identified by outside observers. Other factors that have been associated with sexual violence perpetration, such as psychopathy or pornography use, would be difficult to detect by those who do not know the individual intimately (Abbey et al., 2012; Malamuth et al., 2012). Finally, these three factors were chosen due to their applicability to an on-campus setting. Rape myth acceptance and alcohol consumption tend to be higher in young adults when compared to older adults (Beshers & DiVita, 2021; Boakye, 2009; Schulenberg et al., 2021). Additionally, peer influences are particularly potent, as younger adults tend to value similarities between themselves and their peers more strongly and peer relationships serve an important self-orienting function (Tesch, 1983).

While criminal justice research is important to understand risk identification, there are limitations to extending that research to non-justice-involved campus populations. Research has indicated that risk factors that are prevalent in offender populations may be inappropriately applicable in the context of campus sexual violence, as post-secondary perpetrators have uniquely related factors for risk that are more exclusive to post-secondary populations. Research that is disproportionately focused on offender populations has created a gap of research into campus populations, and only one risk tool for post-secondary settings has been made available (Jung & Mendoza, 2023). Given the prevalence of sexual violence on campus institutions and the low rates of reporting, as well as the fact that a majority of sexual violence research tends to focus on criminal offender populations, greater research is needed to examine how perpetrators of campus sexual assaults are perceived.

## The Current Study

Our current study aimed to examine university student’s perceptions of risk using an experimental design whereby a scenario of campus sexual violence was presented. The scenario was varied to include or exclude the presence of three known risk factors for campus sexual violence perpetration (i.e., rape myth acceptance, alcohol use frequency, and negative peer influence). We sought to understand if students’ perceptions are influenced by these risk factors; specifically, we were interested in their perceptions of an individual who has perpetrated sexual violence in a campus setting. It was hypothesized that students would recognize when an individual who has already done harm is a greater risk when the individual endorses rape myths, frequently uses alcohol, and is exposed to negative peer influences.

## Method

### Participants

This study’s sample comprised 275 undergraduate students at MacEwan University who were recruited from first and second year psychology classes. The mean age of the sample was 20.07 years (*SD* = 3.18) and ranged from 18 to 39 years. The majority were in their first year of study (70.5%, *n* = 191), 19.6% were in their second year of study (*n* = 19.6), 7.7% were in their third year of study (*n* = 21), and 2.2% were in their fourth year (or more) of study (*n* = 6). The sample consisted of 48.3% males (*n* = 130), 50.2% females (*n* = 135), and 1.5% nonbinary/third gender (*n* = 4). Most participants identified their sexuality as heterosexual (84.6%, *n* = 226), while 1.5% indicated homosexual (*n* = 4), 12.4% indicated bisexual or pansexual (*n* = 33), and 1.5% indicated that they were asexual or demisexual (*n* = 4). Participants were 49.8% White/Caucasian (*n* = 124), 4.7% Indigenous (North American Indian, Metis, or Inuit) (*n* = 11), 34.1% Asian (*n* = 85), 3.4% Hispanic/Latino (*n* = 8), 14.4% Black/African (*n* = 34), and 7.8% Middle Eastern (*n* = 18). This current study allowed participants to indicate multiple racial backgrounds; therefore the categories listed are not mutually exclusive.

Current relationship status was also recorded with 4.7% indicating they were married (*n* = 12), 13.6% were in a domestic partnership/civil union (*n* = 35), 5.8% were single and cohabitating with a significant other (*n* = 15), 65.5% were single and never married (*n* = 169), and 10.5% responded that they were dating but not cohabitating (*n* = 27). Finally, we asked participants about any past professional or volunteer experience working with sexual assault victims, and 92.6% reported having no experience (*n* = 252) while only 7.4% indicated they had experience (*n* = 20).

### Materials

A single vignette was created, and various aspects of the vignette were manipulated to reflect the three independent variables examined. Several dependent variables were included to examine perceptions of risk in the form of safety, severity of sanction, reoffending likelihood, and victimization likelihood. Also, questionnaires measuring the participant’s attitudes, beliefs, and demographic information were included to explore any associations with their perceptions of the perpetrator in the vignette. The following describes the vignettes and measures used in this study.

#### Vignettes

Eight conditions of the same vignette were developed to describe an incident of campus sexual violence where the presence of the three independent variables (i.e., rape myth acceptance, frequent use of alcohol, and negative peer influence) was varied. The scenario is presented in second person language, and the participant is asked to place themselves in the scenario as their friend is disclosing the details around a sexual assault she experienced at a campus party. In each vignette, the friend describes a campus party she attends in which she meets and flirts with a male student who eventually asks to speak with her alone. The male student then makes sexual advances that she attempts to resist, but he proceeds anyways and forces sexual contact by touching her genitals. The vignettes depict either the presence or absence of the three risk factors for sexual victimization. These risk factors include rape myth acceptance (i.e., makes statements that endorse rape or does not), frequent use of alcohol (i.e., frequently drinks at campus events or frequency of drinking is not mentioned), and peer support for sexual violence (i.e., has friends who have engaged in sexually inappropriate behaviors or no indication friends engage in such behaviors).

#### Dependent Measures

Four measures were used to examine participants’ perceptions of the perpetrator in the vignette, and are described as follows:

##### Safety Scale (SFT)

Perception of safety was measured using a modified version of Pedneault and Landon’s (2024) 5-item SFT, which is a self-report measure that assesses how safe a participant would feel in different situations with the perpetrator, with each situation varying in degrees of closeness. Our version includes four items, and responses are recorded on a 6-point Likert scale, with higher scores indicating higher feelings of safety around the perpetrator.

##### Severity of Behavior Scale (SBS)

The SBS used in our study is a modified version of Pedneault and Landon’s (2024) severity of punishment subscale and is a 5-item self-report measure that assesses participant’s beliefs that the perpetrator’s actions require various sanctions, and the extent that the behavior constitutes a serious offense. Responses are recorded on a 6-point Likert scale, with higher scores indicating more severe punishment to the perpetrator.

##### Recidivism Scale (RDVM)

The RDVM questions were taken from Pedneault and Landon’s (2024) 5-item subscale of recidivism risk and our version is a 4-item self-report measure that assesses the participant’s perception of the likelihood that the perpetrator will reoffend with new sexual and nonsexual offenses. Responses are recorded on a 6-point Likert scale, with higher scores indicating perceptions that the perpetrator will be more likely to reoffend.

##### Victimization Scale (VCTMZ)

The *VCTMZ* is a 7-item modified version of the 9-item scale from Norris et al.’s study (1999). Our modified version assesses the participant’s level of agreement that the perpetrator will commit various degrees of severity of future sexual victimization. Responses are recorded on a 6-point Likert scale, with higher scores indicating greater likelihood that the perpetrator will commit sexual victimization behaviors.

#### Individual Characteristics

Individual features were also assessed using three measures. Specifically, the participants were asked to complete questionnaires about their sexual experiences, attitudes and beliefs about rape, and views about campus sexual violence prevalence.

##### Sexual Experiences Survey (SES)

The SES (Koss et al., 1987) is a 10-item self-report questionnaire developed to measures the severity of sexual victimization (i.e., unwanted sexual contact, sexual coercion, attempted rape, and rape) experienced since the age of 14. Slight modifications, changing gender references to gender neutral language, were made. Participants completing this questionnaire were asked to choose one of two dichotomous answers (i.e., yes or no) to indicate if they have experienced an event of victimization.

##### Updated Illinois Rape Myth Acceptance Scale (IRMA)

The IRMA (McMahon & Farmer, 2011) is a 19-item self-report questionnaire that assesses the degree to which men and women adhere to rape myths. Participants indicate their agreement with statements on a 6-point Likert scale, with higher scores representing greater endorsement of rape myths.

##### Global Risk Scale (GRS)

The GRS was modified from the scale used in Lee et al.’s (2022) study and is a 5-item self-report questionnaire that measures the participant’s perceptions of the seriousness and frequency of campus sexual violence, and the extent to which campus sexual violence could affect themselves or others. Participants indicate their agreement with statements on a 6-point Likert scale, with higher scores indicating perceptions that campus sexual violence is a very serious problem.

#### Demographic and Post-Manipulation Questionnaires

A self-report questionnaire was used to collect participant’s demographic information. Specifically, participants asked to indicate their age, gender, relationship status, ethnicity, sexual orientation, year in school, and experience working/volunteering with sexual assault victims. Also, a post-manipulation check was conducted using a 5-item self-report questionnaire to assess the participant’s attentiveness to the details within the vignette and assess if participants perceived the events to be sexual assault.

### Procedure

Prior to study administration, ethics approval was sought from the post-secondary institution where participants were recruited. Upon approval, participants signed up for the online study and were redirected to the experiment through the Qualtrics survey platform.

Participants were first presented with a consent form that outlined the purpose and format of the survey, potential risks and benefits, compensation, confidentiality, and the right to withdraw. Then participants were randomly assigned to read one of eight conditions of the vignette where the three independent variables were varied (i.e., rape myth acceptance, frequent attendance at alcohol venues, and negative peer influence). Before reading the vignette, participants were provided with a trigger warning noting that the vignette would include material of a sensitive nature.

Participants were asked to complete the questionnaires capturing the dependent variables, which asked them to rate the likelihood that the respondent would commit another sexual or violent offense, how safe they would feel with the respondent in various contexts, the likelihood that the respondent would commit another act of sexual violence, as well as the severity of the respondent’s behavior. These dependent measures were presented in random order. Then, participants were asked to complete three attitude and beliefs scales, namely the IRMAS, SES, and GRS, which were presented in random order. To ensure the independent variables were salient, participants were asked to complete five post-experiment manipulation check questions, followed by a series of demographic questions. Participants were then debriefed, explaining how and why deception was used, and then finally they were provided a list of local support services as well as researcher contact information. Participants were remunerated with course credit for their participation.

## Results

In order to investigate whether students’ perceptions were influenced by the three risk factors examined in this study, statistical analyses were conducted to examine relationships between the dependent variables and individual characteristics of the sample of university students, and to examine if there were differences between conditions as manipulated through the presentation of the vignette on any of the dependent variables measuring perceptions of perpetrator risk.

First, intercorrelations were calculated for the dependent variables and these are reported in Table 1. Correlations between all dependent variables and covariate measures were calculated using Spearman’s rho. Spearman’s rho rather than Pearson’s *r* was used to calculate these correlations because the risk and sanction appropriateness ratings were ordinal (i.e., numerical scores that fall on an arbitrary numerical scale and are similar to a ranking over a set of points, rather than continuous data). All four dependent measures displayed weak to strong statistically significant (*p* < .01) correlations in both the positive and negative direction, ranging from 0.32 to 0.73. Of the individual characteristics measures (SES, IRMAS, GRS), the IRMAS and GRS were significantly negatively correlated, so the more rape myths endorsed, the less one felt that campus sexual violence was a serious problem. However, IRMAS and SES were not correlated, showing no relationship between rape myth endorsement and past sexual victimization. The SES and GRS had a small but significant positive correlation, suggesting that when there was past sexual victimization, there was also likelihood for endorsing perceptions that campus sexual violence was a problem.

**Table 1:** Intercorrelations Between Dependent Variables and Covariates

Measure	1	2	3	4	5	6
1. SFT Total Score	—	—	—	—	—	—
2. RDVM Total Score	-0.32**	—	—	—	—	—
3. VCTMZ Total Score	-0.31**	0.73**	—	—	—	—
4. SBS Total Score	-0.27**	0.46**	0.61**	—	—	—
5. IRMAS Total Score	0.16	-0.26*	-0.24*	-0.19	—	—
6. SES Total Score	-0.16	0.13	0.11	-0.08	-0.16	—
7. GRS Total Score	-0.24**	0.41**	0.44**	0.28**	-0.30**	0.13

*Note.* SFT = safety scale; RDVM = recidivism scale; VCTMZ = victimization scale; SBS = severity of behavior scale; IRMAS = Illinois Rape Myth Acceptance scale; SES = Sexual Experiences Survey; GRS = global risk scale.

*p < .01. **p < .001.

To examine whether the presence of risk factors (i.e., rape myth acceptance, frequent alcohol use, and negative peer influence) had an impact on students’ perceptions of risk (i.e. perceived safety, severity of behavior, victimization, and recidivism), a multivariate analyses of variance (MANOVA) was conducted. Specifically, factorial 2 X 2 X 2 MANOVAs were carried out to test the combined effects of the independent variables on the dependent variables. The analyses did not reveal statistically significant main effects for rape myth acceptance, Pillai’s Trace, *F*(4,161) = 0.62, *p* = .647, frequent alcohol use, Pillai’s Trace, *F*(4,161) = 1.14, *p* = .338, or negative peer influence, Pillai’s Trace, *F*(4,161) = 2.16, *p* = .076. None of the interactions among the three risk factors were significant (*p* > .05).

To examine whether controlling for the effects of individual characteristics may co-vary with the dependent variables, we conducted separate analyses of covariance (ANCOVAs), specifically entering the total scores for the 3 measures, SES, IRMA, and GRS. We did not find any significant main effects or interactions for each dependent variable (all *p*’s > .05). But for each of the four separate ANCOVAs conducted, global risk perception as measured by the GRS was associated with each dependent variable. GRS was associated with SFT, *F*(1,102) = 13.51, *p* < .001, RDVM, *F*(1,105) = 24.91, *p* < .001, and VCTMZ, *F*(1,111) = 36.59, *p* < .001. This is consistent with the bivariate correlations conducted and reported on Table 1. For the ANCOVA conducted on the dependent variable, SBS measure (indicating beliefs that the individual should require severe sanctions), both global risk perceptions (GBS), *F*(1,107) = 8.27, *p* = .005, and past sexual victimization (SES), *F*(1,107) = 6.19, *p* = .014, were associated with perceptions of punishment, also consistent with the bivariate correlations.

## Discussion

The main objective of this study was to examine the ability of students to identify known risk factors in a scenario of sexual violence in a campus setting using an experimental design to capture variation in their assessment of the perpetrator’s level of risk. Various analogues of risk were used and included perceptions of safety being around the perpetrator, severity of punishment that should be assigned to the perpetrator, the likelihood of committing another offence, and the likelihood of sexually victimizing another person. However, the results of this study suggest that individual or combined risk factors for sexual violence did not significantly affect students’ perceptions of risk, and if the individual who caused harm had all three risk factors of rape myth acceptance, frequent alcohol use, and negative peer influence, this still did not impact a student’s perception of risk. This study was intended to provide insight into what risk factors students may or may not accurately identify and respond to, and our findings suggest that the presence of risk factors did not impact their perceptions of the person who sexually offended.

In addition to examining the ability of students to perceive greater risk through their assessment of a campus sexual violence scenario, we also explored the relationship between students’ individual characteristics (namely, students’ tendency to endorse rape myths, past experiences of sexual victimization, and beliefs about the seriousness of campus sexual violence) and their perceptions of the perpetrator’s level of risk. The tendency to endorse rape myths was associated with perceptions that the perpetrator would be more likely to generally and sexually reoffend. Whereas the tendency to view campus sexual violence as a serious and prevalent problem was associated with lower feelings of safety around the perpetrator, greater perceived likelihood of reoffending—both generally and sexually, and greater perceived seriousness of their behavior that would warrant severe sanctions. Although the presence of the independent variables (i.e., risk factors) showed no significant relationship with perceived risk, students’ global perceptions that campus sexual violence is serious problem was associated with the level of risk the perpetrator posed to others. We were surprised to find that past sexual victimization was not found to be related to any of the dependent variables, acceptance of rape myths, or perceptions of the seriousness of campus sexual violence. Not surprising, endorsement of rape myths was related to lower beliefs that campus sexual violence is a serious concern.

This study provides a preliminary examination of students’ understanding and perceptions of empirically known risk factors. Although these findings are arguably not that distant from research suggesting forensic professionals are not always able to identify risk factors that are related to criminal behavior or sexual violence behavior (see Maltais & Jung, 2019), it is concerning that factors associated with elevated risk for sexual violence do not lead to perceptions that may increase the likelihood of reporting or concerns for safety. There is an increasing awareness of sexual violence on campuses, with many universities and colleges promoting a culture of consent with events, such as sexual violence awareness week or consent awareness week, as well as anti-violence campaigns that have raised awareness (e.g., #metoo, #ibelieveyou, and other campaigns). This growing understanding of campus sexual violence and acceptance that these incidents occur more often than we knew before has led to increased post-secondary resources and implementation of sexual violence policies. But beyond building supports for victims and providing bystander intervention training on campuses, there needs to be a greater awareness in campus communities about what constitutes a greater risk to the community, namely what risk factors are important to consider and assess.

As noted earlier, it is surprising that sexual victimization is not associated with reduced endorsement of rape myths, and it would be relevant to explore whether any other factors may mediate this relationship or if there is a source for rape myth acceptance that may be more impactful in post-secondary settings. It is possible these findings are an artefact of campus communities and student perceptions (Frazier et al., 1995; Zvi & Shechory-Bitton, 2022). Our findings also supported a relationship between perceptions that campus sexual violence is a serious problem and less endorsement of rape myths. Perhaps, knowing the factors that are related to general acceptance of its seriousness may be important to change the minds of administrators to put more emphasis on increasing supports for sexual violence prevention on campuses, addressing the red zone (i.e., a period of time in the school year when there is significantly more incidents of sexual assaults; Follingstad et al., 2023), and ensuring their institutions have sexual violence policies and procedures in place. The obstacle of ‘not believing’ and ‘downplaying the seriousness’ can halt any progress to eliminate sexualized and gender-based violence on campuses and campus communities.

As with any empirical research, our study is not without limitations. It is important to note that only a subset of known sexual violence risk factors were examined, and therefore this experimental design did not include an exhaustive examination of known risk factors. Further study should include other factors that may actually influence student decisions. The current study also recruited from a single academic institution and used a mundane task involving a vignette, hence limiting the study’s generalizability, particularly to students making decisions about their own safety in a real world event (e.g., would they recognize actual risk factors if they were faced with a similar situation, would it change their behavior and response). Our study employed a specific scenario of sexual violence and many sexually violent incidents reported on campuses range from technology-facilitated forms of violence to sexual harassment (Burczycka, 2020). The sample obtained in this study is from an undergraduate institution and therefore a larger sample size that included both undergraduate and graduate students would provide a more diverse representation of the academic community. Last, the vignette used depicted a man assaulting a woman, and therefore, our findings are unlikely to apply to gender diverse scenarios, such as LGBTQ or trans relationships (Fedina et al., 2018).

In conclusion, it is hoped that findings from this study could be used to target appropriate prevention and intervention methods, and perhaps improving recognition of risk factors may lead to enhanced awareness, more effective safety precautions taken, and increased reporting of sexual violence by students, consequently increasing the likelihood for a safer environment for post-secondary institutions and the community.

## Data Availability

The data that supports the findings of this study are available from the corresponding author upon reasonable request.
